# Low-Grade Mucoepidermoid Carcinoma of the Lacrimal Gland in a Teenaged Patient

**DOI:** 10.1155/2017/2418505

**Published:** 2017-11-28

**Authors:** Ozgun Melike Gedar Totuk, Mustafa Kemal Demir, Ozlem Yapicier, Mert Mestanoglu

**Affiliations:** ^1^Department of Ophthalmology, Faculty of Medicine, Bahcesehir University, Istanbul, Turkey; ^2^Department of Radiology, Faculty of Medicine, Bahcesehir University, Istanbul, Turkey; ^3^Department of Pathology, Faculty of Medicine, Bahcesehir University, Istanbul, Turkey; ^4^Bahcesehir University School of Medicine, Istanbul, Turkey

## Abstract

Mucoepidermoid carcinoma is the most common malignant, locally invasive tumour of the salivary glands and accounts for approximately 35% of all malignancies of the major and minor salivary glands. Mucoepidermoid carcinoma that originated from the lacrimal gland is exceedingly rare in teenage patients, with only a few cases reported. Herein, we report clinical and radiological findings of a mucoepidermoid carcinoma arising from the lacrimal gland in a 15-year-old boy. We suggest that since preoperative imaging findings are not diagnostic for mucoepidermoid carcinoma, histopathologic examination should be performed for definitive diagnosis. Complete surgical resection is the treatment of choice for low-grade lacrimal gland mucoepidermoid carcinoma.

## 1. Introduction

Mucoepidermoid carcinoma (MEC), first described by Volkmann in 1895, is the most common malignant epithelial tumour of the salivary glands, accounting for approximately 29–34% of all malignancies of the major and minor salivary glands [1, 2]. The median age at diagnosis is 61 years with a wide range of 8–92 years [1–5]. Unlike salivary glands, MEC of the lacrimal gland is rare (3.6%). MEC involving lacrimal gland is particularly rare among teenage patients with only two cases reported in the English literature [6, 7]. Herein, we report a 15-year-old boy with MEC arising from right lacrimal gland emphasizing magnetic resonance (MR) imaging findings and histopathologic features and discuss differential diagnosis and treatment options.

## 2. Case Presentation

**Figure 1:**
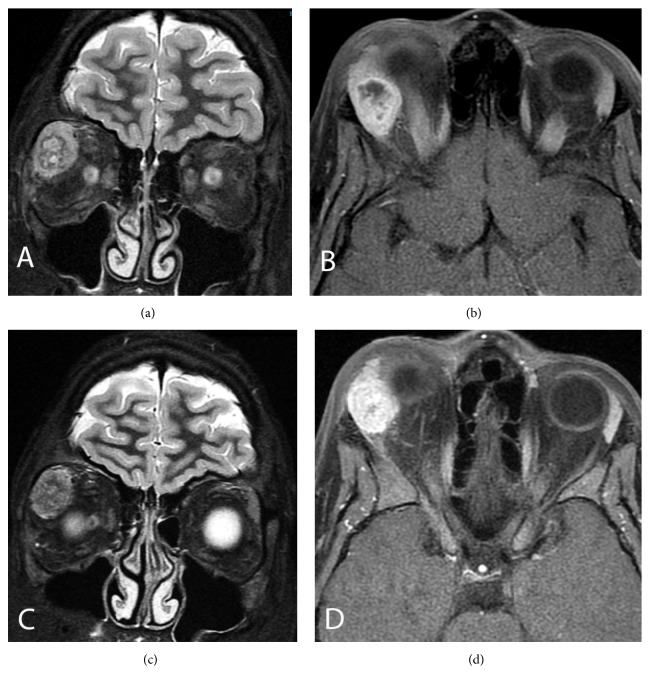
Coronal T2-weighted MR image reveals a heterogeneous oval-shaped right lacrimal gland mass with low and high signals containing solid and cystic components (a). Fat-saturated axial postcontrast MR image obtained at 20 months before the admission shows heterogeneous intense enhanced mass due to unenhancing cystic component (b). Coronal T2-weighted MR image shows a heterogeneous oval-shaped predominantly solid mass with low and high signals without a clear cystic component (c). Fat-saturated axial postcontrast MR image on admission shows almost homogeneous intense enhancement (d).

A 15-year-old boy with a 20-month history of painless right upper eyelid mass and downward displacement of right globe was presented with a recently developed diplopia. On MR images performed 20 months prior to this admission, there had been an irregular oval-shaped soft tissue mass with cystic component in the right lacrimal gland abutting the right globe, and patient had refused the operation at that time. MR images on this admission revealed that tumour was almost replaced by solid component without significant change in size (Figure 1). Tumour was measured 27 mm in diameter and exhibited heterogeneous low and high signals on T2-weighted images, low signals on T1-weighted images, and heterogeneous intense enhancement on postcontrast images. No lymphadenomegaly was detected, and laboratory findings were within normal limits. It was decided that the patient had to be operated on due to recently developed diplopia. Total tumour resection was performed via a lateral orbitotomy with lateral orbital wall removal. Postoperative period was uneventful, and there was no evidence of recurrence at two-year follow-up.

**Figure 2:**
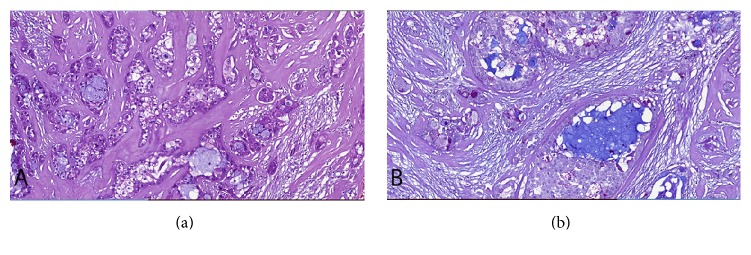
Hematoxylin and eosin stain (original magnification ×20) (a) and periodic acid schiff/alcian blue stain (original magnification ×20) (b) of the tumour specimen showing the pathologic features of a low-grade mucoepidermoid carcinoma of the lacrimal gland.

On gross pathological examination, the tumoural lesion was in a diameter of 25 mm with a cream-colored cut surface. Histopathologically, the tumour was composed of varying proportions of atypical squamous cells, mucus-secreting cells, and intermediate cells forming cords and sheets. Necrosis, neural invasion, and cystic component were not seen. There was also no prominent nuclear atypia or solid pattern. Tumour stroma was hyalinized and sclerotic. Tubuloacinar cells of normal lacrimal gland were seen in the peripheral tissue. There were acute inflammatory cells due to the presence of extracellular mucin released by the rupture of mucus-secreting cells. Mucinous cells were positive for Alcian blue. Reticulogenesis was observed around the tumour cell clusters. Based on these histopathological findings, MEC was diagnosed (Figure 2), and the patient was referred to pediatric oncology. Radiotherapy and chemotherapy were not applied.

## 3. Discussion

The lacrimal gland is a bilobed eccrine secretory gland located in the superotemporal orbit. Most of the neoplasms of the lacrimal gland are originated from epithelial tissues, of which 55% are classified as benign and 45% as malignant [8]. MEC accounts for only 3.6% of all malignant epithelial lacrimal gland tumours [5–10]. In literature, a total of 26 cases with MEC of the lacrimal gland were reported until 2000, among which only one was diagnosed at the age of 12 [6, 7].

Although lacrimal gland tumours may present with various clinical signs and symptoms, majority of patients report facial asymmetry due to eyeball displacement, swelling, decreased eye motility, and rarely diplopia. Most of patients with MEC of lacrimal gland present with isolated, painless, and slow-growing mass with proptosis [9, 11].

The imaging findings of MEC, which are suggestive of preoperative diagnosis, include smooth margins with mucin-containing cystic components, which appear as hyperintense spots on T1- and T2-weighted MR images [12]. However, there may be irregular margins without significant mucinous component on MR imaging as in the presented case. The differential diagnosis of the lacrimal gland MEC on imaging includes inflammatory myofibroblastic tumour, solitary fibrous tumour, alveolar soft part sarcoma, giant cell angiofibroma fibrous histiocytoma, and hemangiopericytoma. Since imaging findings are not characteristic for a definitive diagnosis of MEC, histopathological evaluation is needed.

MEC is usually composed of a mixture of predominantly epidermoid (squamoid) cells, abundant intermediate cells ranging from small basal cells with basophilic cytoplasm to larger cells with eosinophilic cytoplasm, and mucous cells which have a positive PAS reaction by staining with mucicarmine or Alcian blue [13]. Pathological diagnosis of MEC can be difficult due to combination of these three cellular elements in varying proportions sometimes overlapping with benign lesions [14]. The pathological differential diagnosis of MEC of the lacrimal gland may include inverted duct papillomas, cheilitis glandularis, necrotizing sialometaplasia, cystadenoma, cystadenocarcinoma, sebaceous carcinoma and other clear cell tumours, adenosquamous carcinoma, metastatic squamous cell carcinoma, and rarely pleomorphic adenoma.

MECs are histologically graded on the basis of prevalence of mucus cells as low, intermediate, and high-grade. Low-grade tumours are well-differentiated and are made up of over 50% of mucus-secreting elements and squamous epithelial cells. High-grade tumours are poorly differentiated and primarily made up of squamous epithelial and intermediate cells, containing less than 10% of mucus-secreting cells. The histologic features of intermediate-grade tumours fall in between those of the low-grade and high-grade tumours [2, 13, 15]. Histological grading of MEC is most predictive of prognosis and can be used to formulate a therapeutic plan [9].

In the management of low-grade MECs in children, the treatment of choice is complete excision of the tumour with wide margins in order to obtain best outcome. Low to intermediate-grade MECs are more common than high-grade MEC in children; thus radiotherapy or chemotherapy is not recommended [16].

In conclusion, rare incidence and absence of characteristic imaging findings of low-grade lacrimal gland MEC represent a diagnostic challenge and should be considered in the differential diagnosis of lacrimal gland neoplasms in teenage patients. Prognosis is excellent with total tumour resection.
